# Endovascular stroke treatment in orally anticoagulated patients: an analysis from the German Stroke Registry-Endovascular Treatment

**DOI:** 10.1007/s00415-020-10369-6

**Published:** 2020-12-29

**Authors:** Clemens Küpper, Katharina Feil, Frank Arne Wollenweber, Steffen Tiedt, Moriz Herzberg, Franziska Dorn, Thomas Liebig, Marianne Dieterich, Lars Kellert, for the GSR investigators

**Affiliations:** 1 Department of Neurology, Ludwig-Maximilians University, Marchioninistraße 15, 81377 Munich, Germany; 2 German Center for Vertigo and Balance Disorders, Ludwig-Maximilians University, Munich, Germany; 3 Department of Neurology and Stroke, Eberhard-Karls University Tübingen, Universitätsklinikum Tübingen (UKT), Tübingen, Germany; 4 Helios Dr. Horst Schmidt Kliniken Wiesbaden, Wiesbaden, Germany; 5 Institute for Stroke and Dementia Research, University Hospital, Ludwig-Maximilians University, Munich, Germany; 6 Institute of Neuroradiology, Ludwig-Maximilians University, Munich, Germany; 7 Department of Neuroradiology, University of Bonn, Bonn, Germany; 8 Munich Cluster for Systems Neurology (SyNergy), Munich, Germany

**Keywords:** Endovascular treatment, Oral anticoagulation, Large vessel occlusion, Ischemic stroke

## Abstract

**Background:**

Endovascular treatment (ET) in orally anticoagulated (OAC) patients has not been evaluated in randomized clinical trials and data regarding this issue are sparse.

**Methods:**

We analyzed data from the German Stroke Registry-Endovascular Treatment (GSR-ET; NCT03356392, date of registration: 22 Nov 2017). The primary outcomes were successful reperfusion defined as modified thrombolysis in cerebral infarction (mTICI 2b-3), good outcome at 3 months (modified Rankin scale [mRS] 0–2 or back to baseline), and intracranial hemorrhage (ICH) on follow-up imaging at 24 h analyzed by unadjusted univariate and adjusted binary logistic regression analysis. Additionally, we analyzed mortality at 3 months with adjusted binary logistic regression analysis.

**Results:**

Out of 6173 patients, there were 1306 (21.2%) OAC patients, 479 (7.8%) with vitamin K antagonists (VKA) and 827 (13.4%) with non-vitamin K antagonist oral anticoagulation (NOAC). The control group consisted of 4867 (78.8%) non-OAC patients. ET efficacy with the rates of mTICI 2b-3 was similar among the three groups (85.6%, 85.3% vs 84.3%, *p* = 0.93 and 1). On day 90, good outcome was less frequent in OAC patients (27.8%, 27.9% vs 39.5%, *p* < 0.005 and < 0.005). OAC status was not associated with ICH at 24 h (NOAC: odd’s ratio [OR] 0.89, 95% confidence interval [CI] 0.67–1.20; VKA: OR 1.04, CI 0.75–1.46).

Binary logistic regression analysis revealed no influence of OAC status on good outcome at 3 months (NOAC: OR 1.25, CI 0.99–1.59; VKA: OR 1.18, CI 0.89–1.56) and mortality at 3 months (NOAC: OR 1.03, CI 0.81–1.30; VKA: OR 1.04, CI 0.78–1.1.37).

**Conclusions:**

ET can be performed safely and successfully in LVO stroke patients treated with OAC.

**Clinical trial registration-URL:**

http://www.clinicaltrials.gov. Unique identifier: NCT03356392.

**Supplementary Information:**

The online version contains supplementary material available at 10.1007/s00415-020-10369-6.

## Introduction

Stroke treatment for large vessel occlusion (LVO) strokes has been revolutionized in 2015 by the publication of the so-called HERMES trials (*Highly Effective Reperfusion Evaluated in Multiple Endovascular Stroke Trials*) [3, 4, 6, 8, 18]. Since then, endovascular treatment (ET) is the standard of care in LVO ischemic strokes apart from intravenous thrombolysis (IVT). Nevertheless, it remains unclear whether ET is safe and efficacious in certain patient subgroups. In particular, ET in OAC stroke patients has not been evaluated formally in the HERMES trials. OAC—variably defined—was even an exclusion criterion in some of them. [3, 4, 8, 18] Since then, several mostly small retrospective and four prospective observational studies investigated ET in anticoagulated patients [2, 5, 9–17, 19, 21, 22]. Because of the overwhelming success of ET, large randomized-controlled trials on this topic are not to be expected in the future due to ethical concerns. Therefore, we evaluated the safety and efficacy of ET for LVO stroke in a large cohort of OAC patients compared to non-OAC patients within the prospectively collected data of the German Stroke Registry-Endovascular Treatment (GSR-ET).

## Materials and methods

### Study cohort

The study cohort consisted of patients included to the GSR-ET, an academic, prospective, multicenter registry study for the systematic evaluation of the outcome, safety, and process parameters of ET in standard of care in Germany. It has been described in depth recently [1, 20]. In brief, all consecutive adult patients with LVO stroke with an intention to be treated with ET at 25 German study centers were included to the GSR-ET. For this current evaluation, all GSR-ET patients were analyzed who were either orally anticoagulated or not anticoagulated at the time of stroke based on the local neurologist’s evaluation of the patient’s medication history and laboratory results if performed locally. Laboratory investigations such as INR or anti factor Xa activity, etc. were not systematically performed and documented in the registry to confirm the medication history. Patients without information on anticoagulation therapy were excluded from the analysis.

### Data collection

Source data were collected at 25 stroke centers in Germany between June 2015 and December 2019, and were assessed and rated by the local neurointerventionalists and neurologists. Codified data were stored in a web-based electronic database hosted at the University Medical Center Hamburg-Eppendorf.

### Efficacy and safety outcomes

The primary efficacy outcomes were the successful recanalization defined as modified thrombolysis in cerebral infarction (mTICI) score 2b-3 analyzed by the local neurointerventionalist and the modified Rankin scale (mRS) after 3 months analyzed by the local neurologist either during an outpatient visit or by phone call if the patient could not come to the outpatient clinic. Good outcome at 3 months was defined according to mRS 0–2 or back to baseline to account for possible pre-stroke disability. The primary safety outcomes were presence of intracranial hemorrhage (ICH) on routine follow-up imaging at 24 h according to the ECASS II (European Cooperative Acute Stroke Study [part II]) definition [7], irrespective of the presence of new clinical symptoms and without registry documentation of the specific ICH subtype. A secondary analysis included the prediction of good outcome on the mRS at 3 months, death at 3 months and ICH on follow-up imaging at 24 h by the anticoagulation status adjusted for age, sex, comorbidities, concomitant antiplatelet therapy, stroke severity, and concomitant IVT therapy.

### Statistical analysis

Data are presented as mean ± standard deviation (SD) or counts and percentages as applicable. Data were analyzed by Kruskal–Wallis test, chi-squared test, or Fisher’s exact test as applicable. A Bonferroni correction for multiple testing was used. Differences were accepted as statistically significant for the following *p* values: *p* < 0.05, *p* < 0.01 and *p* < 0.005. For secondary analysis, a binary logistic regression analysis adjusting for potential confounder variables was performed with 95% confidence intervals (CI). Potential confounder variables were determined from meaningful baseline between-group differences of univariate analysis and chosen based on clinical experience (IVT, smoking, atrial fibrillation, diabetes mellitus, arterial hypertension, antiplatelet therapy, sex, age, NIHSS on admission, and pmRS). Additionally, for mRS at d90, an ordinal regression shift analysis was performed. All tests were performed with IBM® SPSS^®^ Statistics Version 26.

## Results

### Baseline patient characteristics

In total, 6636 patients were analyzed of whom 6173 patients fulfilled the inclusion criteria. 1306 (21.2%) patients were treated with OAC: 479 (7.8%) patients had OAC with vitamin K antagonists (VKA) and 827 (13.4%) patients with non-vitamin K antagonist oral anticoagulants (NOAC). The control group consisted of 4867 (78.8%) non-OAC patients. OAC patients were older (VKA 77.7 ± 10.9 years, NOAC 77.7 ± 10.9 years vs no OAC 72.0 ± 13.5 years, *p* < 0.005) and more NOAC patients were female (52.6% and 57.8% vs 49.5%, *p* = 0.11 and < 0.005). Concomitant antiplatelet therapy was less frequent among OAC patients (5.6% and 9.4% vs 37.4%, *p* < 0.005). As expected, OAC patients had more often atrial fibrillation (86.9% and 87.5% vs 29.9%, *p* < 0.005) but also arterial hypertension (88.4% and 86.4% vs 75.3%, *p* < 0.005). Also, among OAC patients, diabetes mellitus (25.2% and 28.4% vs 20.7%, *p* = 0.05 and < 0.005) and hyperlipidemia (48.4% and 45.5% vs 37.9%, *p* < 0.005) were more frequent, while smoking was less prevalent (17.5% and 19.4% vs 27.3%, *p* < 0.005). Premorbid functional status analyzed by the premorbid mRS was worse in the OAC patients (median 0 and 0 vs 0, interquartile range 2 and 2 vs 1, *p* < 0.005) (see Table 1).

**Table 1:** Baseline patient characteristics

Parameter	No OAC	VKA	*p* value	NOAC	*p* value	Included patients, *n* (%)
*n* (%)	4867 (78.8)	479 (7.8)	n. a	827 (13.4)	n. a	6173 (100)
Age, mean ± SD	72.0 ± 13.5	77.7 ± 10.9	< 0.005	77.7 ± 10.9	< 0.005	6169 (99.9)
Female, *n* (%)	2409 (49.5)	252 (52.6)	0.11	469 (57.8)	< 0.005	6170 (100)
Antiplatelet therapy, *n* (%)	1819 (37.4)	27 (5.6)	< 0.005	78 (9.4)	< 0.005	6173 (100)
Arterial hypertension, *n* (%)	3632 (75.3)	419 (88.4)	< 0.005	703 (86.4)	< 0.005	6112 (99.0)
Diabetes mellitus, *n* (%)	998 (20.7)	119 (25.2)	0.05	231 (28.4)	< 0.005	6107 (98.9)
Hyperlipidemia, *n* (%)	1823 (37.9)	228 (48.4)	< 0.005	368 (45.5)	< 0.005	6093 (98.7)
Atrial fibrillation, *n* (%)	1438 (29.9)	413 (86.9)	< 0.005	715 (87.5)	< 0.005	6107 (98.9)
History of smoking, *n* (%)	1208 (27.3)	72 (17.5)	< 0.005	144 (19.4)	< 0.005	5568 (90.2)
pmRS, median (min, max; interquartile range)	0 (0, 5; 1)	0 (0, 5; 2)	< 0.005	0 (0, 5; 2)	< 0.005	6005 (97.3)

*OAC* oral anticoagulation, *VKA* vitamin K antagonist, *NOAC* non-vitamin K antagonist oral anticoagulant, *pmRS* premorbid modified Rankin scale

### Stroke, imaging, and treatment characteristics

Clinical stroke severity analyzed by the National Institute of Health Stroke Scale (NIHSS) showed more severe strokes in OAC patients (15 and 15 vs 14, *p* = 0.05 and < 0.005). The Alberta Stroke Program Early CT Score (ASPECTS) was equally distributed among the three groups (9 and 9 vs 9, *p* = 0.79 and 1). Occlusion site of the LVO was similar in all three groups except for extracranial Carotid artery occlusion which was less frequent among NOAC patients (2.6% vs 6.9% in non-OAC patients, *p* < 0.005). Also, multiple LVO and occlusion sides were similarly distributed among orally anticoagulated patients and the control group. As expected, IVT was less frequently performed in OAC patients (26.7% and 11.0% vs 59.4%, *p* < 0.005). Whether IVT in these OAC patients was performed because of subtherapeutic anticoagulation or because these patients were misclassified as non-OAC patients at the time of IVT initiation remains speculative as the GSR-ET did not collect data on this question. If performed on site, IVT was started less fast in OAC than in non-OAC patients (door-to-needle time in minutes 38 ± 33 and 46 ± 26 vs 33 ± 49, *p* < 0.005), while in drip-and-ship patients, door-to-needle time was not different (− 93 ± 118 and − 72 ± 60 vs − 87 ± 75, *p* = 0.28 and 0.72). ET was initiated (door-to-groin puncture time in minutes 89 ± 133 and 97 ± 156 vs 101 ± 151, *p* = 0.21 and 0.13) and terminated (door-to-flow restoration time in minutes 137 ± 148 and 137 ± 149 vs 139 ± 134, *p* = 1 and 0.19) similarly fast in OAC compared to non-OAC patients. With regards to stroke etiology, cardioembolic stroke was more frequent among OAC patients both VKA and NOAC patients (88.9% and 83.8% vs 40.7%, *p* < 0.005). Large artery atherosclerosis (4.4% and 4.7% vs 30.1%, *p* < 0.005) and undetermined cause (3.3% and 6.2% vs 23.3%, *p* < 0.005) were less frequent among OAC patients. Also, other determined causes were less frequent among VKA patients (3.3% vs 5.9%, *p* = 0.04), while they were similarly frequent among NOAC patients (5.3% vs 5.9%, *p* = 1) (see Table 2).

**Table 2:** Clinical stroke, imaging, and treatment characteristics

Parameter	No OAC	VKA	*p* value	NOAC	*p* value	Included patients, *n* (%)
NIHSS, median (min, max)	14 (0, 42)	15 (0, 42)	0.05	15 (0, 42)	< 0.005	6078 (98.5)
ASPECTS, median (min, max)	9 (1, 10)	9 (1, 10)	0.79	9 (1, 10)	1	4830 (78.2)
Occlusion site, *n* (%)						
Carotid artery extracranial	333 (6.9)	22 (4.6)	0.11	21 (2.6)	< 0.005	6094 (98.7)
Carotid artery intracranial without Carotid-T	261 (5.4)	26 (5.5)	1	30 (3.7)	0.08	
Carotid artery intracranial including Carotid-T	746 (15.5)	91 (19.1)	0.08	135 (16.5)	1	
M1 proximal	1629 (33.9)	142 (29.8)	0.15	280 (34.3)	1	
M1 distal	977 (20.3)	93 (19.5)	1	161 (19.7)	1	
M2	982 (20.5)	100 (21.0)	1	172 (21.1)	1	
ACA	104 (2.2)	18 (3.8)	0.07	22 (2.7)	0.74	
PCA	122 (2.5)	14 (2.9)	1	26 (3.2)	0.69	
BA	485 (10.1)	35 (7.4)	0.13	71 (8.7)	0.46	
VA	96 (2.0)	8 (1.0)	0.10	5 (1.1)	0.32	
Occlusion side						
Right, *n* (%)	2114 (43.4)	200 (41.8)	1	355(42.9)	1	6173 (100)
Left, *n* (%)	2333 (47.9)	255 (53.2)	0.06	418 (50.5)	0.35	
n. a., e.g., BA, *n* (%)	492 (10.1)	34 (7.1)	0.07	72 (8.7)	0.46	
Multiple, *n* (%)	101 (2.1)	10 (2.1)	1	21 (2.5)	0.87	
IVT yes, *n* (%)	2892 (59.4)	128 (26.7)	< 0.005	91 (11.0)	< 0.005	6173 (100)
IVT yes and on site, *n* (%)	1674 (34.4)	55 (6.7)	< 0.005	72 (15.0)	< 0.005	6173 (100)
Door-to-needle time IVT on site, minutes, mean ± SD	33 ± 49	38 ± 33	< 0.005	46 ± 26	< 0.005	1686 (93.6)
Door-to-needle time IVT drip-and-ship, minutes, mean ± SD	− 87 ± 75	− 93 ± 118	0.28	− 72 ± 60	0.72	1310 (88.6)
Door-to-groin puncture time, mean ± SD	101 ± 151	89 ± 133	0.21	97 ± 156	0.13	5827 (94.4)
Door-to-flow restoration time, mean ± SD	139 ± 134	137 ± 148	1	137 ± 149	0.19	5091 (82.4)
Stroke etiology, *n* (%)						
Cardioembolic	1982 (40.7)	426 (88.9)	< 0.005	693 (83.8)	< 0.005	6173 (100)
Large artery atherosclerosis	1466 (30.1)	21 (4.4)	< 0.005	39 (4.7)	< 0.005	
Other determined cause	285 (5.9)	16 (3.3)	0.04	44 (5.3)	1	
Undetermined cause	1134 (23.3)	16 (3.3)	< 0.005	51 (6.2)	< 0.005	

*OAC* oral anticoagulation, *VKA* vitamin K antagonist, *NOAC* non-vitamin K antagonist oral anticoagulant, *NIHSS* National Institute of Health Stroke Scale, *ASPECTS* Alberta Stroke Program Early CT Score, *ACA* anterior cerebral artery, *PCA* posterior cerebral artery, *BA* basilar artery, *VA* vertebral artery, *IVT* intravenous thrombolysis, *SD* standard deviation

### Efficacy and safety of ET

**Fig. 1:**
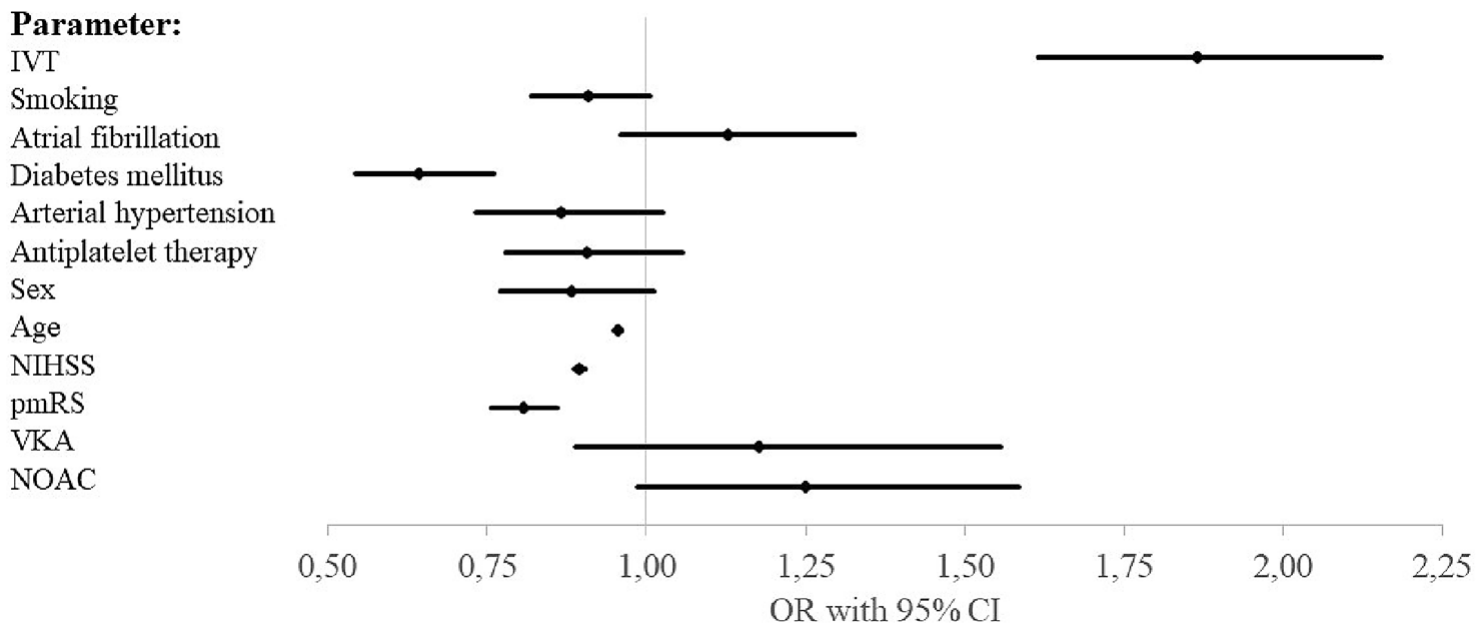
Binary logistic regression analysis for good outcome (mRS 0–2 or back to baseline) at d90. *mRS* modified Rankin scale, *IVT* intravenous thrombolysis, *NIHSS* National Institute of Health Stroke Scale, *pmRS* premorbid modified Rankin scale, *VKA* vitamin K antagonist, *NOAC* non-vitamin K oral anticoagulation anticoagulant

**Fig. 2:**
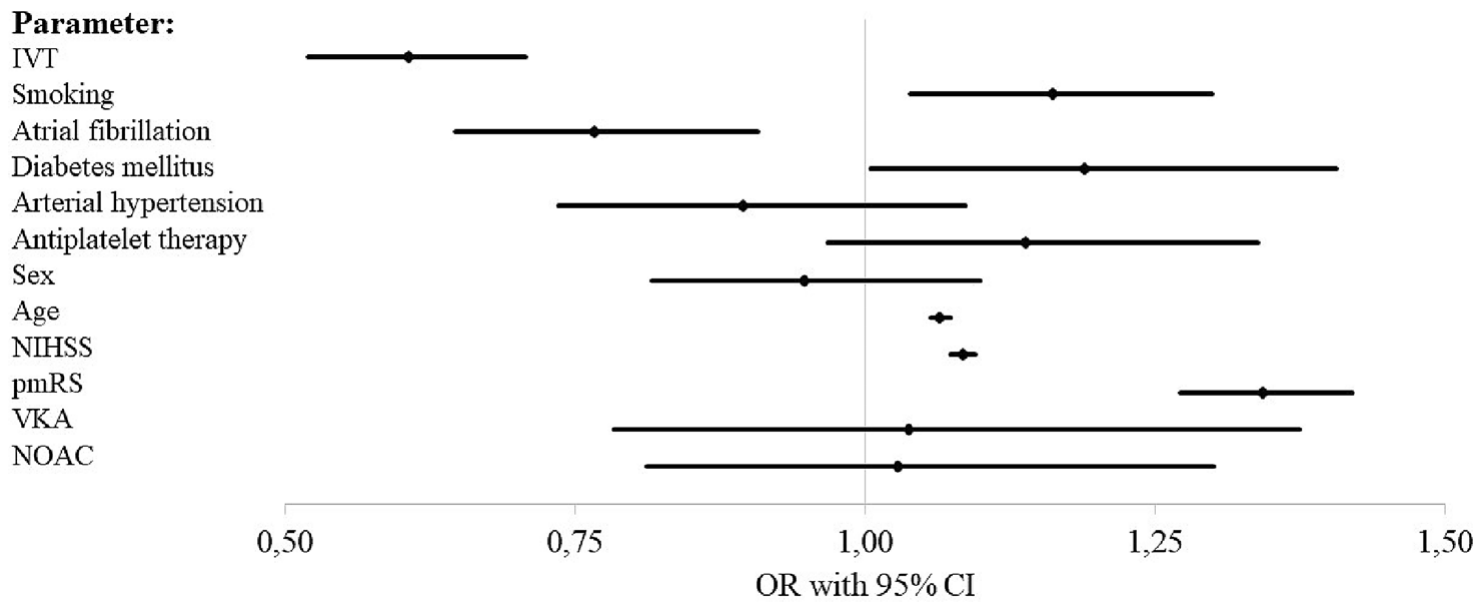
Binary logistic regression analysis for mortality at d90. *IVT* intravenous thrombolysis, *NIHSS* National Institute of Health Stroke Scale, *pmRS* premorbid modified Rankin scale, *VKA* vitamin K antagonist, *NOAC* non-vitamin K oral anticoagulant

**Fig. 3:**
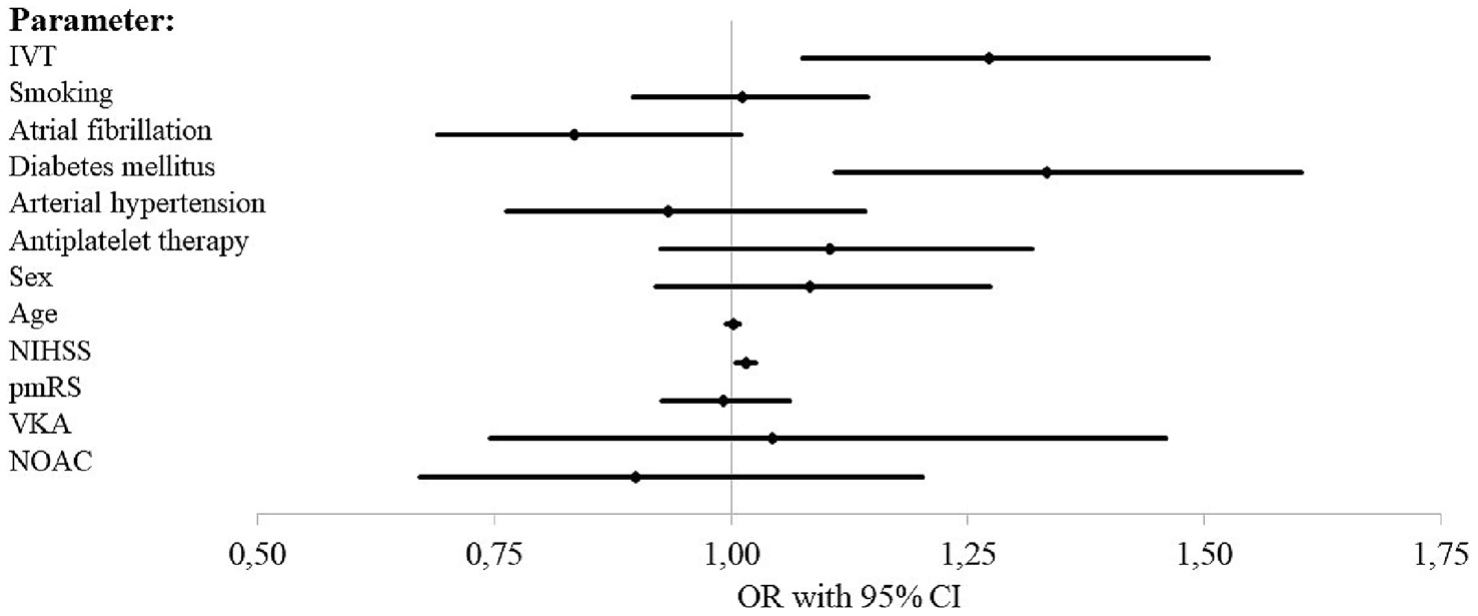
Binary logistic regression analysis for ICH at 24 h. *ICH* intracranial hemorrhage, *IVT* intravenous thrombolysis, *NIHSS* National Institute of Health Stroke Scale, *pmRS* premorbid modified Rankin scale, *VKA* vitamin K antagonist, *NOAC* non-vitamin K oral anticoagulant

Successful reperfusion (mTICI 2b-3) after ET was achieved with equal frequency among orally anticoagulated patients (85.6% and 85.3% vs 84.3%, *p* = 0.93 and 1) (see Table 3). While on day 90, good outcome was less frequent in OAC patients in univariate analysis (31.3%, 31.1% vs 41.4%, *p* < 0.005) (see Table 3) binary logistic regression analysis with adjustment for pmRS, NIHSS, age, sex, antiplatelet medication, arterial hypertension, diabetes mellitus, atrial fibrillation, smoking, and IVT performed revealed no statistically significant influence of oral anticoagulation status on good outcome at 3 months (NOAC: odd’s ratio [OR] 1.25, confidence interval [CI] 0.99–1.586; VKA: OR 1.18, CI 0.89–1.56) (see Fig. 1 and electronic supplemental Table 1). This finding was confirmed with an ordinal regression shift analysis of the mRS at d90 which did not reveal a statistically significant influence of OAC status on the mRS shift (NOAC: OR 0.90, CI 0.75–1.09; VKA: OR 0.93, CI 0.75–1.15) (see electronic supplemental Table 2). The same was true for mortality at 3 months (NOAC: OR 1.03, CI 0.81–1.30; VKA: OR 1.04, CI 0.78–1.37) (see Fig. 2 and electronic supplemental Table 3). With regards to safety, in the majority of the patients, ICH at 24 h was determined on CT scans (*n* = 5079, 82%), while MRI only was used in a minority of 618 patients (10%). A small subgroup of 229 patients (3.7%) was investigated with CT and MRI scans. Neither univariate analysis nor binary logistic regression analysis revealed a statistically significant influence of the oral anticoagulation status on ICH on follow-up imaging at 24 h (univariate: *p* = 0.44 and 0.06; binary logistic regression: NOAC: OR 0.90, CI 0.67–1.20; VKA: OR 1.04, CI 0.75–1.46) (see Table 3, Fig. 3 and electronic supplemental table 4). Interestingly, within the VKA and the NOAC group IVT did not increase the risk for ICH at 24 h in univariate analysis (VKA: ICH at 24 h and IVT vs no IVT: 12.5% vs 12.5%, *p* = 1.0; NOAC: ICH at 24 h and IVT vs no IVT: 11.0% vs 11.8%, *p* = 1.0).

**Table 3:** Safety and efficacy outcomes in univariate analysis

Parameter	No OAC	VKA	*p* value	NOAC	*p *value	Included patients, *n* (%)
Reperfusion rate (mTICI 2b–3), *n* (%)	4032 (84.3)	404 (85.6)	0.93	701 (85.3)	1	6077 (98.4)
ICH 24 h, *n* (%)	712 (14.6)	60 (12.5)	0.44	97 (11.7)	0.06	6173 (100)
mRS 0–2 on d90, *n* (%)	1672 (39.5)	116 (27.8)	< 0.005	197 (27.9)	< 0.005	5353 (86.7)
Good outcome (mRS 0–2 or back to baseline) on d90, *n* (%)	1744 (41.4)	130 (31.3)	< 0.005	218 (31.1)	< 0.005	5332 (86.4%)

*OAC* oral anticoagulation, *VKA* vitamin K antagonist, *NOAC* non-vitamin K antagonist oral anticoagulation anticoagulant, *mTICI* modified thrombolysis in cerebral infarction, *ICH* intracranial hemorrhage, *mRS* modified Rankin scale

## Discussion

The key findings of our retrospective analysis of a large German prospectively collected observational cohort of LVO stroke patients who received ET in view of their OAC status are as follows: ET in LVO OAC patients is effective and safe with regards to reperfusion rate and postinterventional ICH. Moreover, OAC status did not have a negative influence on the long-term functional outcome after ET. Although LVO OAC patients performed worse on the mRS at 3 months, their OAC status did not affect the rate of good functional outcome and mortality at 3 months after adjustment for possible confounding variables. The poorer performance of OAC patients on the mRS at 3 months might at least be in part explained by their worse premorbid functional status (higher premorbid mRS), their older age, and the more sever strokes in NOAC patients (higher NIHSS). All three of these variables had a negative impact on the rate of good outcome at 3 months in the multivariate analysis. In addition, comorbidities were more frequent among OAC patients. In contrast, differences in the pre-interventional imaging on admission as a possible confounder for the clinical efficacy of ET in OAC patients can be excluded as ASPECTS on admission and type and side of LVO were in general comparable among OAC and non-OAC LVO stroke patients. Also, ET was initiated and finished equally fast in OAC and non-OAC patients. This excludes a prolongation of the procedural times in non-OAC patients due to higher rates of IVT or in OAC patients due to a more difficult decision process before performing ET and/or IVT which could have had an impact on the technical and clinical outcomes.

Our analysis comprises the highest number of patients with 6173 in total and 1306 OAC patients compared to the recently published studies with a total number of patients ranging from 28 to 1913 and a number of OAC patients ranging from 26 to 320 [2, 5, 9–17, 19, 21, 22]. Most studies published retrospectively collected data, but four studies were prospective cohort studies [2, 5, 9–17, 19, 21, 22]. With regards to technical efficacy of ET, if reported, all studies showed similar reperfusion rates in OAC and non-OAC patients as in our GSR-ET cohort [2, 5, 9, 11, 16, 19, 21, 22]. Nevertheless, the reperfusion rates were distributed heterogeneously among the different studies ranging from 64 to 93% [2, 5, 9, 11, 16, 19, 21, 22]. Additionally, one study compared ET in VKA and NOAC patients and reported on better reperfusion rates among NOAC patients [12] which was not reflected by our data showing similar reperfusion rates among OAC and non-OAC patients. Clinical efficacy of ET in OAC patients compared to non-OAC patients analyzed by the mRS at 3 months was reported to be good in most of the recently published studies [2, 9, 11, 16, 17, 21, 22]. Nevertheless, one Czech study found that an mRS 0–2 on day 90 seemed to be less frequent among patients on anticoagulation therapy, but those patients were older and had more comorbidities [5]. Similarly, another study reported worse outcome on d90 among patients on VKA, but again this turned out to be mainly attributable to higher age [19]. Supporting these observations, in our own analysis after adjustment for possible confounding factors including age and comorbidities, neither NOAC nor VKA treatment did have a negative impact on the long-term clinical efficacy of ET. With regards to mortality, one French study reported a higher mortality rate at three months in anticoagulated patients anticoagulated with VKA, NOAC, or heparin [11]. Another recent multicenter observational cohort study published together with a study-level meta-analysis found an increased mortality at 90 days specifically for VKA patients but not for NOAC patients receiving MT [13]. In general, other published studies [2, 5, 9, 16, 21, 22] and our own data did not support this observation: in our patients, neither VKA nor NOAC intake on admission increased mortality on d90 after MT. These findings on the mortality rate are in accordance with the published ICH rates as a safety parameter that did not seem to be higher among the OAC patients as in our cohort although reported differentially detailed [2, 5, 9–11, 14, 16, 17, 19, 21, 22]. Interestingly, the multicenter observational cohort study that had already reported on increased mortality among VKA patients also found increased rates of symptomatic intracranial hemorrhage among these patients which might at least explain the higher mortality rates [13]. Our own study did not see higher ICH rates among VKA and NOAC patients, although our data are not directly comparable as we evaluated any ICH on 24 h imaging that means not only symptomatic but also asymptomatic.

In summary, our analysis of the GSR-ET as the largest available cohort supports the general impression from the literature that ET is safe and efficacious in OAC patients. In particular, long-term functional outcome and mortality were not influenced by OAC status. Additionally, intracranial hemorrhage rates were not increased.

Nevertheless, our analysis has several limitations: first, our data are of observational character. Therefore, generalizability has to be assumed with caution only. Yet, given the overwhelming success of ET in LVO strokes, it will be difficult to generate randomized-controlled data on this topic in the future due to ethical concerns which underscores the importance of large retrospective analyses such as ours. Second, the OAC status of our patients was not verified by laboratory results such as INR, specific drug activity measurement, etc. as they were not broadly documented in our study database. This might have led to misclassification of some patients. Third, data on some parameters were missing which might have influenced our interpretation of the results. Fourth, ICH on postinterventional imaging was not further characterized in the registry which limits the interpretation of the safety of ET in OAC patients. Additionally, in a majority of the patients, hemorrhagic transformation at 24 h was determined by CT scans without further distinguishing between dual-energy and ordinary CT scan within the registry. CT scans at 24 h might not be able to differentiate fairly enough between ICH and postinterventional blood–brain barrier disruption (BBRD) compared to imaging at 72 h or alternative imaging modalities such as MRI.

## Conclusion

ET in OAC patients with acute LVO ischemic strokes should be strongly considered as it is a safe and efficacious treatment.

## Supplementary Information

Below is the link to the electronic supplementary material.

Supplementary file1 (DOCX 30 KB)

## Data Availability

The data that support the findings of this study are available from the corresponding author upon reasonable request.
