# Supplementary material for: Endovascular stroke treatment in orally anticoagulated patients: an analysis from the German Stroke Registry-Endovascular Treatment
Source: J Neurol. 2020 Dec 29;268(5):1762–9. doi: 10.1007/s00415-020-10369-6 (PMC8068620; doi:10.1007/s00415-020-10369-6)
Supplement: Supplementary file 1 — Supplementary file1 (DOCX 30 KB) [file 415_2020_10369_MOESM1_ESM.docx]

**Electronic supplemental material**

**Article title:**

Endovascular stroke treatment in orally anticoagulated patients: An analysis from the German Stroke Registry-Endovascular Treatment

**Journal name:**

Journal of Neurology

**Authors:**

Clemens Küpper, MD; Katharina Feil, MD, Frank Arne Wollenweber, MD; Steffen Tiedt, MD, PhD; Moriz Herzberg, MD; Franziska Dorn, MD; Thomas Liebig, MD, Marianne Dieterich, MD, Lars Kellert, MD, for the GSR investigators

**Corresponding author:**

PD Dr. Lars Kellert

Department of Neurology, Ludwig Maximilians University

E-Mail: [Lars.Kellert@med.uni-muenchen.de](mailto:Lars.Kellert@med.uni-muenchen.de)

**Supplemental table 1:** Binary logistic regression analysis for good outcome (mRS 0-2 or back to baseline) at d90

| **Parameter** | **Odd’s ratio** | **95% confidence interval** | **p-value** | **Full data set**, n (%) |
| --- | --- | --- | --- | --- |
| NOAC on admission | 1.25 | 0.99-1.59 | 0.07 | 4733 (76.7) |
| VKA on admission | 1.18 | 0.90-1.56 | 0.26 |  |
| pmRS | 0.81 | 0.76-0.86 | <0.005 |  |
| NIHSS on admission | 0.90 | 0.89-0.91 | <0.005 |  |
| Age | 0.96 | 0.95-0.96 | <0.005 |  |
| Sex | 0.88 | 0.77-1.01 | 0.07 |  |
| Antiplatelet therapy | 0.91 | 0.78-1.06 | 0.21 |  |
| Arterial hypertension | 0.87 | 0.73-1.03 | 0.10 |  |
| Diabetes mellitus | 0.64 | 0.54-0.76 | <0.005 |  |
| Atrial fibrillation | 1.13 | 0.96-1.33 | 0.14 |  |
| Smoking | 0.91 | 0.82-1.01 | 0.07 |  |
| IVT | 1.87 | 1.62-2.15 | <0.005 |  |

Abbr.: mRS modified Rankin scale, NOAC non-vitamin K oral anticoagulant, VKA vitamin K antagonist, pmRS premorbid modified Rankin scale, NIHSS National Institute of Health Stroke Scale, IVT intravenous thrombolysis

Supplemental table 2: Ordinal regression analysis for mRS at d90

| **Parameter** | **Odd’s ratio** | **95% confidence interval** | **p-value** | **Full data set**, n (%) |
| --- | --- | --- | --- | --- |
| NOAC on admission | 0.90 | 0.75-1.09 | 0.28 |  |
| VKA on admission | 0.93 | 0.75-1.15 | 0.50 |  |
| pmRS | 1.48 | 1.41-1.55 | <0.005 |  |
| NIHSS on admission | 1.10 | 1.09-1.11 | <0.005 |  |
| Age | 1.05 | 1.04-1.05 | <0.005 |  |
| Sex | 1.05 | 0.95-1.17 | 0.34 |  |
| Antiplatelet therapy | 1.13 | 1.00-1.34 | 0.05 |  |
| Arterial hypertension | 1.01 | 0.88-1.15 | 0.92 |  |
| Diabetes mellitus | 1.42 | 1.25-1.62 | <0.005 |  |
| Atrial fibrillation | 0.84 | 0.74-0.95 | <0.01 |  |
| Smoking | 1.14 | 1.00-1.29 | <0.05 |  |
| IVT | 0.57 | 0.51-0.64 | <0.005 |  |

Abbr.: ICH intracranial hemorrhage, NOAC non-vitamin K oral anticoagulant, VKA vitamin K antagonist, pmRS premorbid modified Rankin scale, NIHSS National Institute of Health Stroke Scale, IVT intravenous thrombolysis

**Supplemental table 3:** Binary logistic regression analysis for mortality at d90

| **Parameter** | **Odd’s ratio** | **95% confidence interval** | **p-value** | **Full data set**, n (%) |
| --- | --- | --- | --- | --- |
| NOAC on admission | 1.03 | 0.81-1.30 | 0.82 | 4750 (76.9) |
| VKA on admission | 1.04 | 0.78-1.37 | 0.80 |  |
| pmRS | 1.34 | 1.27-1.42 | <0.005 |  |
| NIHSS on admission | 1.08 | 1.07-1.10 | <0.005 |  |
| Age | 1.06 | 1.06-1.07 | <0.005 |  |
| Sex | 0.95 | 0.82-1.10 | 0.47 |  |
| Antiplatelet therapy | 1.14 | 0.97-1.34 | 0.12 |  |
| Arterial hypertension | 0.89 | 0.74-1.09 | 0.12 |  |
| Diabetes mellitus | 1.19 | 1.01-1.41 | <0.05 |  |
| Atrial fibrillation | 0.77 | 0.65-0.91 | <0.005 |  |
| Smoking | 1.16 | 1.04-1.30 | <0.01 |  |
| IVT | 0.61 | 0.52-0.71 | <0.005 |  |

Abbr.: NOAC non-vitamin K oral anticoagulant, VKA vitamin K antagonist, pmRS premorbid modified Rankin scale, NIHSS National Institute of Health Stroke Scale, IVT intravenous thrombolysis

**Supplemental table 4:** Binary logistic regression analysis for ICH 24h

| **Parameter** | **Odd’s ratio** | **95% confidence interval** | **p-value** | **Full data set**, n (%) |
| --- | --- | --- | --- | --- |
| NOAC on admission | 0.90 | 0.67-1.20 | 0.90 | 5387 (87.3) |
| VKA on admission | 1.04 | 0.75-1.46 | 0.80 |  |
| pmRS | 0.99 | 0.93-1.06 | 0.99 |  |
| NIHSS on admission | 1.02 | 1.01-1.03 | <0.005 |  |
| Age | 1.00 | 1.00-1.009 | 0.54 |  |
| Sex | 1.08 | 0.92-1.27 | 0.33 |  |
| Antiplatelet therapy | 1.11 | 0.93-1.32 | 0.23 |  |
| Arterial hypertension | 0.93 | 0.76-1.14 | 0.50 |  |
| Diabetes mellitus | 1.33 | 1.11-1.60 | <0.005 |  |
| Atrial fibrillation | 0.84 | 0.69-1.01 | 0.84 |  |
| Smoking | 1.01 | 0.90-1.14 | 0.843 |  |
| IVT | 1.27 | 1.08-1.50 | <0.005 |  |

Abbr.: ICH intracranial hemorrhage, NOAC non-vitamin K oral anticoagulant, VKA vitamin K antagonist, pmRS premorbid modified Rankin scale, NIHSS National Institute of Health Stroke Scale, IVT intravenous thrombolysis

**Appendix**

GSR Investigators

| Name | Degree | Organization | Role | Contribution | Email |
| --- | --- | --- | --- | --- | --- |
| Tobias Boeckh-Behrens | MD | Klinikum r.d.Isar, Munich, Germany | Site Investigator | German Stroke Registry - Steering Committee | boeckh-behrens@tum.de |
| Silke Wunderlich | MD | Klinikum r.d.Isar, Munich, Germany | Site Investigator | German Stroke Registry - Steering Committee | silke.wunderlich@tum.de |
| Alexander Ludolph | MD | Sana Klinikum Offenbach | Site Investigator | German Stroke Registry - Steering Committee | alexander.ludolph@sana.de |
| Karl-Heinz Henn | MD | Sana Klinikum Offenbach | Site Investigator | German Stroke Registry - Steering Committee | Karl-Heinz.Henn@sana.de |
| Arno Reich | MD | Uniklinik RWTH Aachen, Germany | Site Investigator | German Stroke Registry - Steering Committee |  |
| Anastasios Mpotsaris | MD | Uniklinik RWTH Aachen, Germany | Site Investigator | German Stroke Registry - Steering Committee |  |
| Martin Wiesmann | MD | Uniklinik RWTH Aachen, Germany | Site Investigator | German Stroke Registry - Steering Committee |  |
| Ulrike Ernemann | MD | Tübingen University Hospital, Germany | Site Investigator | German Stroke Registry - Steering Committee | Ulrike.Ernemann@med.uni-tuebingen.de |
| Sven Poli | MD | Tübingen University Hospital, Germany | Site Investigator | German Stroke Registry – Steering Committee | Sven.poli@uni-tuebingen.de |
| Christian H Nolte | MD | Charite Campus Benjamin Franklin | Site Investigator | German Stroke Registry - Steering Committee | Christian.Nolte@charite.de |
| Eberhard Siebert | MD | Charité – Campus Benjamin Franklin und Campus Charité Mitte, Berlin, Germany | Site Investigator | German Stroke Registry - Steering Committee | Eberhard.siebert@charite.de |
| Sarah Zweynert | MD | Charité - Campus Virchow Klinikum, Berlin, Germany | Site Investigator | German Stroke Registry - Steering Committee | sarah.zweynert@charite.de |
| Georg Bohner | MD | Charité - Campus Virchow Klinikum, Berlin, Germany | Site Investigator | German Stroke Registry - Steering Committee | Georg.bohner@charite.de |
| Laszlo Solymosi | MD | Uniklinik Bonn | Site Investigator | German Stroke Registry - Steering Committee | laszlo.solymosi@ukbonn.de |
| Gabor Petzold | MD | Uniklinik Bonn | Site Investigator | German Stroke Registry - Steering Committee | gabor.petzold@ukb.uni-bonn.de |
| Waltraud Pfeilschifter | MD | Uniklinik Frankfurt/ Main, Germany | Site Investigator | German Stroke Registry - Steering Committee | Waltraud.Pfeilschifter@kgu.de |
| Fee Keil | MD | Uniklinik Frankfurt/ Main, Germany | Site Investigator | German Stroke Registry - Steering Committee | Fee.Keil@kgu.de |
| Joachim Röther | MD | Asklepios Klinik Altona, Hamburg, Germany | Site Investigator | German Stroke Registry - Steering Committee | j.roether@asklepios.com |
| Bernd Eckert | MD | Asklepios Klinik Altona, Hamburg, Germany | Site Investigator | German Stroke Registry - Steering Committee | b.eckert@asklepios.com |
| Jörg Berrouschot | MD | Klinikum Altenburger Land, Altenburg, Germany | Site Investigator | German Stroke Registry - Steering Committee | joerg.berrouschot@klinikum-altenburgerland.de |
| Albrecht Bormann | MD | Klinikum Altenburger Land, Altenburg, Germany | Site Investigator | German Stroke Registry - Steering Committee | albrecht.bormann@klinikum-altenburgerland.de |
| Anna Alegiani | MD | University Medical Center Hamburg-Eppendorf, Hamburg, Germany | Site Investigator | German Stroke Registry - Steering Committee | a.alegiani@uke.de |
| Jens Fiehler | MD | University Medical Center Hamburg-Eppendorf, Hamburg, Germany | Site Investigator | German Stroke Registry - Steering Committee | fiehler@uke.de |
| Christian Gerloff | MD | University Medical Center Hamburg-Eppendorf, Hamburg, Germany | Site Investigator | German Stroke Registry - Steering Committee | gerloff@uke.de |
| Götz Thomalla | MD | University Medical Center Hamburg-Eppendorf, Hamburg, Germany | Site Investigator | German Stroke Registry - Steering Committee | thomalla@uke.de |
| Sven Thonke | MD | Klinikum Hanau, Germany | Site Investigator | German Stroke Registry - Steering Committee |  |
| Christopher Bangard | MD | Klinikum Hanau, Germany | Site Investigator | German Stroke Registry - Steering Committee |  |
| Christoffer Kraemer | MD | Klinikum Lüneburg, Germany | Site Investigator | German Stroke Registry - Steering Committee | christoffer.kraemer@klinikum-lueneburg.de |
| Martin Dichgans | MD | Ludwig Maximilian University of Munich, Germany | Site Investigator | German Stroke Registry - Steering Committee | ‎Martin.Dichgans@med.uni-muenchen.de |
| Marios Psychogios | MD | Georg-August-Universität Göttingen, Germany | Site Investigator | German Stroke Registry - Steering Committee |  |
| Jan Liman | MD | Georg-August-Universität Göttingen, Germany | Site Investigator | German Stroke Registry - Steering Committee |  |
| Martina Petersen | MD | Klinikum Osnabrück, Germany | Site Investigator | German Stroke Registry - Steering Committee | Martina.Petersen@klinikum-os.de |
| Florian Stögbauer | MD | Klinikum Osnabrück, Germany | Site Investigator | German Stroke Registry - Steering Committee | florian.stoegbauer@klinikum-os.de |
| Peter Kraft | MD | University Hospital Würzburg, Germany | Site Investigator | German Stroke Registry - Steering Committee |  |
| Mirko Pham | MD | University Hospital Würzburg, Germany | Site Investigator | German Stroke Registry - Steering Committee |  |
| Michael Braun | MD | Bezirkskrankenhaus Günzburg, Germany | Site Investigator | German Stroke Registry - Steering Committee | Michael.Braun@bkh-guenzburg.de |
| Gerhard F. Hamann | MD | Bezirkskrankenhaus Günzburg, Germany | Site Investigator | German Stroke Registry - Steering Committee | gerhard.hamann@bkh-guenzburg.de |
| Christian Roth | MD | Klinikum Bremen Mitte, Germany | Site Investigator | German Stroke Registry - Steering Committee |  |
| Klaus Gröschel | MD | University Medical Center Mainz, Germany | Site Investigator | German Stroke Registry - Steering Committee | Klaus.Groeschel@unimedizin-mainz.de |
| Timo Uphaus | MD | University Medical Center Mainz, Germany | Site Investigator | German Stroke Registry - Steering Committee | Timo.Uphaus@unimedizin-mainz.de |
| Volker Limmroth | MD | Kliniken Köln, Germany | Site Investigator | German Stroke Registry - Steering Committee |  |
